# Guided Bone Regeneration for Vertical Alveolar Augmentation: Clinical Evaluation of d-PTFE-Ti Membranes and Comparison with Collagen Membranes: A Systematic Review

**DOI:** 10.3390/jfb17080353

**Published:** 2026-07-23

**Authors:** Chloé Duchatelle, Rosana Costa, Ana Sofia Vinhas, Filomena Salazar, Cristina Cabral, Cátia Reis

**Affiliations:** 1 Faculty of Dental Medicine, University of Health Science (IUCS-CESPU), 4585-116 Gandra, Portugal; a31904@alunos.cespu.pt; 2 Department of Medicine and Oral Surgery, University of Health Science (IUCS-CESPU), 4585-116 Gandra, Portugal; ana.vinhas@iucs.cespu.pt (A.S.V.); filomena.salazar@iucs.cespu.pt (F.S.); cristina.cabral@iucs.cespu.pt (C.C.); catia.reis@iucs.cespu.pt (C.R.); 3 Oral Pathology and Rehabilitation Research Unit (UNIPRO), University of Health Science (IUCS-CESPU), 4585-116 Gandra, Portugal

**Keywords:** guided bone regeneration, vertical ridge augmentation, d-PTFE membrane, collagen membrane

## Abstract

Based on the growing use of guided bone regeneration (GBR) for implant rehabilitation in atrophic alveolar ridges, the choice of barrier membrane remains a critical factor for clinical success. This review aimed to evaluate the clinical effectiveness of titanium-reinforced dense polytetrafluoroethylene (d-PTFE-Ti) membranes compared with resorbable collagen membranes in vertical ridge augmentation. A literature review was conducted using PubMed, Cochrane Library, and ScienceDirect databases. Clinical studies published since 2014 involving human subjects undergoing GBR procedures with either d-PTFE-Ti or collagen membranes were included. Nine studies met the inclusion criteria, including randomized clinical trials and cohort studies. The selected studies indicated that both membrane-based approaches were effective in promoting bone regeneration and enabling implant placement. Titanium-reinforced d-PTFE membranes may offer mechanical advantages in space maintenance and dimensional stability, particularly in larger vertical defects; however, the available comparative evidence did not demonstrate a consistent statistically significant superiority over collagen-based approaches. Within the limitations of the available evidence, both strategies appear to provide clinically predictable outcomes, and membrane selection should be individualized according to defect morphology and treatment requirements.

## 1. Introduction

The volumetric alteration of the maxillary and mandibular bone is a critical consequence of tooth loss, limiting rehabilitation with dental implants. Among the available techniques, guided bone regeneration (GBR) has become a widely documented and effective method for ridge augmentation, particularly in cases of significant vertical bone loss. The biological principle of GBR is based on the selective exclusion of epithelial and connective tissue cells, allowing osteogenic cells to colonize the defect and promote new bone formation [1]. The underlying biological mechanisms of GBR involve the creation and maintenance of a protected space, stabilization of the blood clot, and adequate vascularization of the graft, key factors for the formation of new bone [1]. The choice of membrane plays a central role in this process, as it directly influences the stability of the regenerative space, the predictability of the outcome, and the complication profile [2]. Various biomaterials have been studied to optimize periodontal and bone regeneration, with particular attention to the interaction between grafts and barrier membranes [3]. According to the literature, there are two main types of membranes: resorbable membranes, usually collagen-based, and non-resorbable membranes, such as d-PTFE, each with distinct characteristics in terms of stability, resorption time, and space-maintaining capacity [2,3]. The first non-resorbable membranes used clinically were expanded polytetrafluoroethylene (e-PTFE) membranes, with an open fibrous structure, which have the longest published follow-up data and are considered the “gold standard” for the treatment of advanced defects [4]. More recently, dense polytetrafluoroethylene (d-PTFE) membranes have been introduced; their compact structure offers greater resistance to bacterial penetration in cases of exposure, although it results in reduced tissue interaction with the membrane surface. Clinical studies demonstrate that the use of d-PTFE was effective in maintaining space and protecting the graft, promoting predictable bone gains in complex vertical augmentation cases. Vertical alveolar ridge augmentation presents additional challenges, as it requires greater graft stability and strict control of soft tissue invasion. Modern techniques, including the use of titanium-reinforced PTFE meshes, have shown promising results, with consistent vertical bone gains and an acceptable complication rate [5]. Despite the widespread use of resorbable collagen-based membranes, these have limitations in vertical procedures, mainly due to early resorption and a reduced capacity to maintain the space required for significant bone regeneration [5]. In this context, the aim of conducting the present study is, on one hand, the need to clarify the available scientific evidence regarding the clinical effectiveness of titanium-reinforced d-PTFE membranes in vertical bone augmentation procedures. Despite the growing number of publications in this field, the literature presents heterogeneous results regarding evaluation methods, follow-up periods, complication rates, and comparisons with resorbable collagen-based membranes. It therefore becomes relevant to gather, analyze, and critically discuss the current evidence in order to support therapeutic decision-making and the selection of the most appropriate biomaterial according to the characteristics of the bone defect. Thus, this systematic review aims to evaluate the clinical effectiveness and associated complications of the use of titanium-reinforced dense polytetrafluoroethylene (d-PTFE-Ti) membranes in guided bone regeneration (GBR) procedures for vertical augmentation of the alveolar ridge, predominantly in implant-related contexts. The secondary objective consists of comparing the clinical and regenerative performance of these d-PTFE-Ti membranes with that of resorbable collagen-based membranes in vertical bone augmentation. Beyond their barrier function, membrane selection is influenced by the biological and mechanical requirements of the defect. Recent reviews emphasize that an ideal GBR membrane should combine biocompatibility, cell occlusiveness, space maintenance, adequate handling, and controlled tissue integration. Non-resorbable PTFE-based barriers provide high structural stability, whereas collagen membranes offer favorable biocompatibility and avoid a second surgical procedure for membrane removal; however, their mechanical properties and degradation behavior may limit space maintenance in demanding defects [6,7,8]. Recent developments in resorbable membranes have therefore focused on cross-linking, multilayer structures, functional coatings, and other modifications intended to improve mechanical stability, degradation control, and biological activity [7,9]. In parallel, current biological research highlights that GBR outcomes depend not only on passive cell exclusion but also on the local immune environment. Macrophages may contribute to vascularization, osteogenic signaling, biomaterial degradation, and the transition from inflammation to tissue regeneration. The crosstalk between macrophages and mesenchymal stem cells may further coordinate inflammatory resolution and osteogenic processes during bone healing, supporting the relevance of membrane–host interactions in successful regeneration [10,11].

## 2. Materials and Methods

This systematic review was conducted and reported in accordance with the Preferred reporting Items for systematic Reviews and Meta-Analyses (PRISMA 2020) [12].

The study methodology, literature search, study selection process, data extraction, and reporting of results were performed following the PRISMA 2020 guidelines. The completed PRISMA 2020 Checklist is provided as Supplementary Materials Table S1.

### 2.1. PICOS’ Question

The following guiding question was defined according to the study design, population, intervention, comparison and outcomes: “What is the clinical effectiveness and what are the complications associated with the use of titanium-reinforced d-PTFE membranes in guided bone regeneration procedures for vertical augmentation of the alveolar ridge, compared with collagen-based resorbable membranes?”. The study protocol for this systematic review was registered on the International Prospective of Systematic Reviews (PROSPERO), under number CRD420261422623. Table 1 shows the ‘PICOS’ strategy used.

### 2.2. Search Strategy

The literature search was conducted using the PubMed (via the National Library of Medicine), Cochrane, and ScienceDirect databases. The literature search was conducted between December 2024 and April 2025, with the final search performed in April 2025.

Studies published between 2014 and 2025 were considered eligible. This timeframe was predefined to focus on contemporary clinical evidence related to guided bone regeneration techniques and currently relevant membrane-based approaches for vertical alveolar ridge augmentation.

This study provides a fundamental contribution to the comparative assessment of the clinical and radiographic performance of GBR membranes, directly aligned with the primary objective of this systematic review, namely the evaluation of the clinical effectiveness and associated complications of d-PTFE-Ti membranes. It also provides essential comparative data for the secondary analysis contrasting these membranes with resorbable collagen membranes. The following keywords were used for the search: “guided bone regeneration”, “vertical ridge augmentation”, “d-PTFE membrane”, and “collagen membrane”.

### 2.3. Search Terms

An advanced search was performed in PubMed, Cochrane, and ScienceDirect using combinations of MeSH terms with an 11-year time restriction:

- ((guided bone regeneration) AND (vertical ridge augmentation OR membrane OR biomaterial))
- ((guided bone regeneration) AND (vertical ridge augmentation)) OR ((vertical ridge augmentation) AND (d-PTFE-Ti)) OR ((vertical ridge augmentation) AND (collagen membrane))
- (titanium reinforced PTFE OR d-PTFE OR resorbable collagen membrane) AND (vertical ridge augmentation) AND (randomized clinical trial)
- (“vertical ridge augmentation”) AND (“Polytetrafluoroethylene” OR d-PTFE OR “dense PTFE” OR “titanium-reinforced d-PTFE” OR “non-resorbable PTFE membrane”) AND (“Collagen”OR “collagen membrane”).

Table 2 shows the inclusion criteria employed.

### 2.4. Study Selection

After removing duplicate articles, the initial stage of study selection was carried out by screening the titles and abstracts of the articles. Studies that did not meet the eligibility criteria were excluded. In the second stage of selection, the same eligibility criteria were applied to the remaining studies in full-text form. The study selection process was performed independently by two reviewers to enhance the reliability and reproducibility of the screening process. A two-stage screening method was used to ensure a rigorous and reliable selection of eligible studies. In the first stage, the two independent reviewers screened the titles and abstracts of all identified records according to the predefined inclusion and exclusion criteria. Each reviewer independently determined whether each record should be included, excluded, or retained for full-text assessment. In the second stage, the same two reviewers independently assessed the full texts of the studies that passed the initial screening against the predefined eligibility criteria. Any discrepancies between the two reviewers were resolved through discussion and, when consensus could not be reached, by consultation with a third reviewer.

### 2.5. Risk of Bias and Quality Assessment

The risk of bias analysis showed variability in the methodological quality of the included studies. Most randomized clinical trials presented a low to moderate overall risk of bias. The risk of bias assessment was performed using the Joanna Briggs Institute (JBI) guidance 2017 for each type of study (randomized controlled trials and cohort studies) (Table 3 and Table 4). The Joanna Briggs Institute (JBI) critical appraisal tools were selected because the included studies comprised different methodological designs, including randomized controlled trials and cohort studies. Questionnaire items in each type of research questionnaire were answered with the responses ‘Yes’ (Y), ‘No’ (N) or ‘Uncertain’ (UN). Unlike the Cochrane RoB 2 tool, which is specifically designed for randomized trials, the JBI provides design-specific appraisal checklists, allowing each study to be assessed according to its methodological characteristics within a consistent critical appraisal framework.

## 3. Results

### 3.1. Search Results

**Figure 1:**
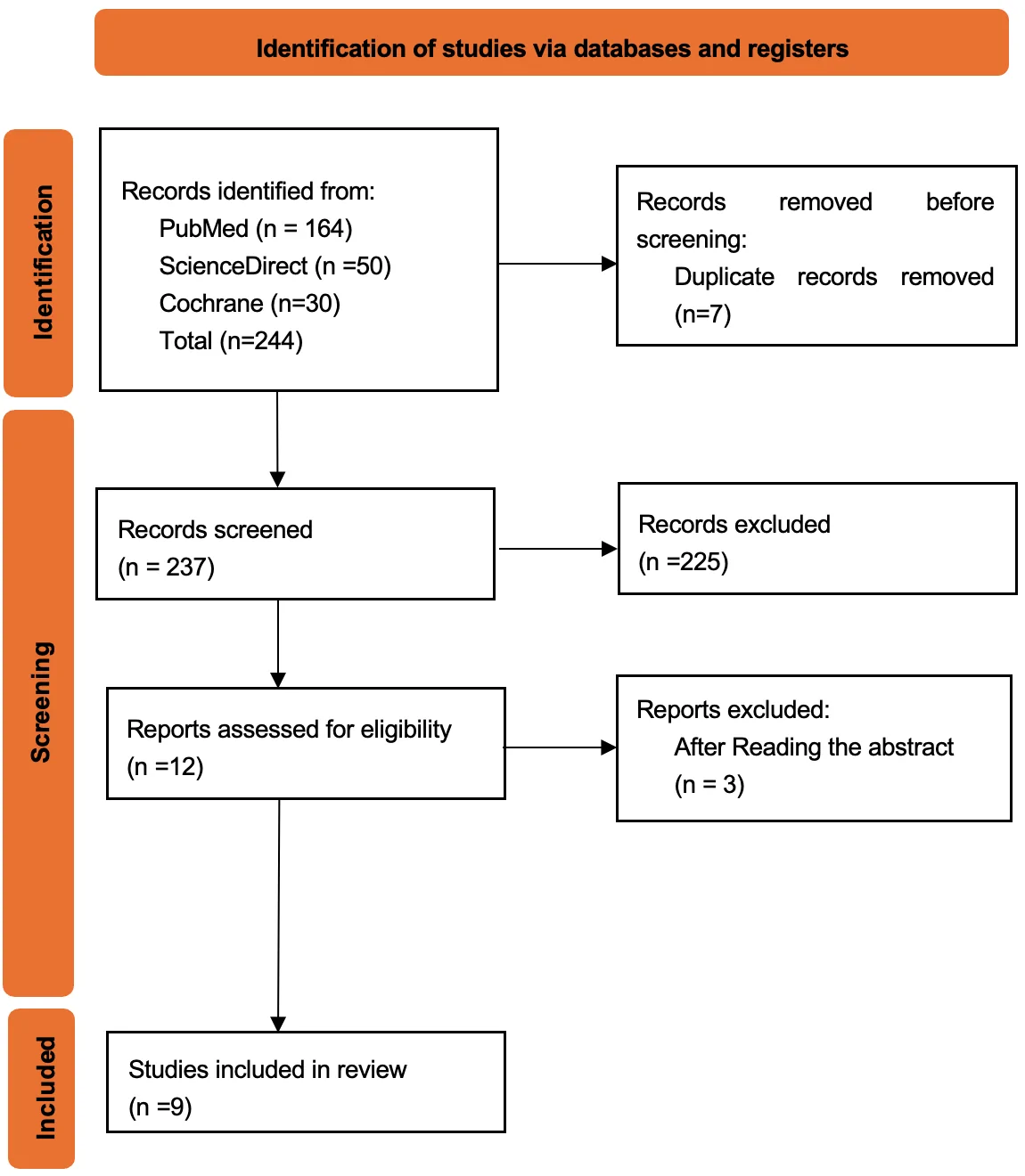
PRISMA flowchart summarizing the study identification and selection process.

A total of 244 studies were initially identified. After removal of duplicates, records were screened based on titles and abstracts. Finally, 9 studies met the inclusion criteria and were included in the systematic review, all of which underwent full-text analysis, as illustrated in Figure 1.

### 3.2. Analysis of the Included Studies

A total of 9 clinical studies met the final eligibility criteria after excluding three studies that did not meet the final eligibility criteria (Arbab 2020, Mandarino 2018, and Scheyer 2016). The final selection included eight randomized clinical trials and one cohort study, published between 2014 and 2025, evaluating guided bone regeneration procedures relevant to vertical alveolar ridge augmentation. Sample sizes varied across studies, generally ranging from 22 to 40 patients. The included studies evaluated titanium-reinforced non-resorbable barriers, resorbable collagen membranes, titanium meshes associated with collagen membranes, and other membrane-based regenerative approaches. Several publications represented different analyses or follow-up periods of the same clinical trial; therefore, the number of participants reported across individual publications was not summed to avoid double-counting overlapping patient populations. Overall, the included studies provided a clinically relevant evidence base for evaluating regenerative outcomes, complication profiles, and the comparative performance of non-resorbable and resorbable membrane-based approaches in vertical ridge augmentation procedures.

### 3.3. Characterization of Sample Bias Risk

Quality assessments are shown in Table 1 for randomized controlled trials and Table 4 for cohort studies. The degree of quality of the studies on the relational index used and the number of positive responses to the questions are mostly high. Table 5 shows the main characteristics of the included studies.

**Table 5:** The main characteristics of the included studies.

Study	Design/Follow-Up	Sample	Intervention/Comparison	Main Outcomes	Main Findings
Merli et al. (2014) [19]	Randomized Clinical trial; 6 years	22 patients	Resorbable collagen barrier vs. titanium-reinforced non-resorbable barrier	Bone-level changes	Mean bone level at 6 years: - 1.33 mm in the resorbable group (RES) - 1.00 mm in the non-resorbable group (N-RES) Adjusted between-group difference: 0.15 mm (*p* = 0.5713); no statistically significant difference in long-term bone stability.
Cucchi et al. (2017) [13]	Randomized Clinical trial	40 patients	Ti-reinforced d-PTFE membrane vs. Ti-mesh + collagen membrane	Vertical bone gain; complications; implants osseointegration	Similar vertical bone gain (4.2 ± 1.0 vs. 4.1 ± 1.0 mm); no statistically significant between-group difference.
Park et al. (2017) [21]	Cohort Study	32 patients	Collagen membrane combined with three different grafting materials	Vertical bone gain; dimensional changes	Vertical bone gain ranged from 3.90 to 5.13 mm according to graft type; sufficient bone volume was maintained for implant placement.
Cucchi et al. (2019) [16]	Randomized Clinical trial; histological analysis	40 patients	Ti-reinforced d-PTFE membrane vs. Ti-mesh + collagen membrane	Newly formed bone; residual biomaterial; soft tissue	Similar newly formed bone (39.7% vs. 42.1%); no statistically significant between-group difference.
Cucchi et al. (2021) [14]	Randomized Clinical trial; 1 year	40 patients	Ti-reinforced d-PTFE membrane vs. Ti-mesh + collagen membrane	Marginal bone loss; complications	Similar marginal bone loss (0.67 vs. 0.61 mm) and comparable complication rates.
Cucchi et al. (2023) [15]	Randomized Clinical trial; 3 year	28 patients/79 implants	Ti-reinforced d-PTFE membrane vs. Ti-mesh + collagen membrane	Vertical bone gain; marginal bone loss; complications	Similar vertical bone gain (4.2 ± 1.0 vs. 4.1 ± 1.0 mm) and marginal bone loss (<1 mm); implant survival was 100%.
Giragosyan et al. (2024) [20]	Randomized Clinical trial	40 patients	Custom 3D-printed Ti-mesh vs. Ti-reinforced d-PTFE membrane	Linear bone gain; healing complications	Bone gain: 3.65 ± 1.73 vs. 4.24 ± 2.19 mm; complication rates were comparable between groups
Strauss et al. (2025) [18]	Randomized Clinical trial; 6 month	25 patients	Resorbable collagen membrane vs. Ti-reinforced e-PTFE membrane	Buccal bone reduction; soft-tissue thickness	Significantly less buccal bone reduction with the non-resorbable membrane (0.1 ± 0.4 vs. 0.8 ± 0.4 mm).
Urban et al. (2025) [17]	Randomized Clinical trial	30 patients	Ti-reinforced PTFE mesh alone vs. the same mesh + collagen membrane	Vertical bone gain; regeneration rate; complications	Absolute vertical bone gain: - PTFE-Ti group: 4.47 ± 2.05 mm - PTFE-Ti + collagen membrane group: 4.11 ± 2.69 mm Similar vertical bone gain and complication rates; adding collagen did not significantly improve outcomes.

## 4. Discussion

The results of this review suggest that guided bone regeneration (GBR) can provide clinically relevant outcomes for alveolar ridge augmentation, particularly in the vertical dimension, thereby creating favorable conditions for implant-based rehabilitation. The available evidence indicates that both titanium-reinforced non-resorbable membranes and collagen-based resorbable approaches can promote bone regeneration, although differences may exist in their clinical behavior, stability, and complication profiles.

These findings are consistent with recent reviews of GBR biomaterials, which describe membrane performance as a balance between barrier function, mechanical stability, tissue integration, and clinical handling rather than as a single universally superior material characteristic [6,8]. Evidence syntheses focused on vertical bone regeneration similarly suggest that different membrane systems can support clinically relevant bone gain, while the certainty of direct comparisons remains limited by heterogeneity in techniques, defect characteristics, and outcome assessment [22].

From a biological perspective, the recent literature also suggests that regenerative outcomes are influenced by immune–stromal interactions within the healing environment. Macrophage polarization and macrophage–mesenchymal stem cell crosstalk may affect inflammatory resolution, vascularization, and osteogenic signaling, providing a biological framework for understanding why membrane properties and local tissue responses can influence bone regeneration [10,11].

In the studies included in this systematic review, several authors evaluated the performance of titanium-reinforced d-PTFE membranes compared with other barriers used in GBR. Randomized clinical trials reported that these membranes were capable of maintaining the regenerative space and achieving clinically relevant vertical bone gains, particularly in atrophic alveolar ridge areas intended for implant placement [15,17,18,19]. The vertical bone gain values reported in these studies support the clinical applicability of vertical augmentation techniques and highlight the importance of membrane stability and graft protection during the healing period.

Beyond vertical bone gain itself, some studies also assessed the medium- and long-term stability of regenerated bone. For example, long-term follow-up studies have shown that peri-implant bone levels can remain relatively stable over the years following initial regeneration [13,18]. These findings suggest that regeneration achieved with titanium-reinforced d-PTFE membranes not only increases bone volume but may also provide adequate structural stability for implant maintenance.

On the other hand, several studies have directly compared non-resorbable membranes with resorbable collagen membranes. Overall, the results indicate that both can achieve comparable levels of bone regeneration, although differences may exist regarding surgical handling, complication rates, and biological behavior during healing [14,15,17,21]. In particular, histological and histomorphometric studies have shown that regenerated areas may present similar percentages of newly formed bone between different membrane types, confirming the overall effectiveness of guided bone regeneration approaches [16].

Another relevant aspect observed in the studies analyzed concerns the complications associated with GBR procedures. Early membrane exposure is one of the most frequently reported complications in the literature and may partially compromise the regenerative process. However, several clinical trials indicate that titanium-reinforced d-PTFE membranes can present acceptable complication rates and, in some cases, even lower rates than those observed with other augmentation techniques, such as the use of titanium meshes [15,18,19].

More recent investigations have explored new approaches and materials to optimize bone regeneration outcomes, including the use of customized titanium meshes or the combination of different biomaterials [18]. These advances reflect the ongoing evolution of guided bone regeneration techniques and the search for solutions that improve clinical predictability, reduce complications, and maximize new bone formation. Therefore, it is important to further analyze in detail the main aspects related to the clinical outcomes obtained.

### 4.1. Clinical Effectiveness of d-PTFE-Ti Membranes in Vertical Bone Augmentation

The available evidence suggests that guided bone regeneration using titanium-reinforced dense polytetrafluoroethylene (d-PTFE-Ti) membranes can achieve clinically relevant outcomes in vertical alveolar ridge augmentation. The studies included in this review reported effective maintenance of the regenerative space and stabilization of the bone graft, with vertical bone gains that enabled subsequent implant placement.

Several randomized clinical trials have directly analyzed vertical bone gain achieved with d-PTFE-Ti membranes. In the study conducted by Cucchi et al. [15], which compared d-PTFE-Ti membranes with titanium meshes combined with collagen membranes, a similar mean vertical bone gain was observed between the two groups, with values close to 4 mm of vertical augmentation. These results indicate that both approaches were effective for vertical regeneration of the alveolar ridge. Although a lower surgical complication rate was reported in the d-PTFE-Ti group in this individual study, vertical bone gain was comparable between the two groups. This finding should therefore be interpreted as a potential difference in the complication profile rather than evidence of overall clinical superiority.

Comparable results were also observed in the study by Cucchi et al. [14], a subsequent work from the same group with a 1-year follow-up. In this study, the authors also reported vertical bone gains exceeding 4 mm, with no statistically significant differences between d-PTFE-Ti membranes and titanium meshes covered with collagen membranes. In addition, good stability of peri-implant tissues was observed, with marginal bone loss of less than 1 mm during the first year, indicating that the regenerated bone was able to adequately support the placed implants.

Long-term stability of the outcomes was confirmed in the study by Cucchi et al. [15] with a 3-year follow-up. The authors observed that the initial vertical bone gains remained stable over time, with mean values close to 4–5 mm of vertical augmentation in both the d-PTFE-Ti group and the titanium mesh with collagen membrane group. These findings support the medium-term stability of the regenerated bone.

Similar results were also described in the more recent randomized clinical trial conducted by Giragosyan et al. [20], which compared the use of d-PTFE-Ti membranes with customized 3D-printed titanium meshes. The authors observed comparable linear bone gains between the two groups, demonstrating that d-PTFE-Ti membranes can provide similar regenerative outcomes even when compared with advanced digitally designed rigid space-maintaining devices.

Although several studies included in this review focus specifically on vertical alveolar ridge augmentation, other works evaluated parameters related to bone stability. For example, the long-term study by Merli et al. [19], with a 6-year follow-up, analyzed changes in bone levels after vertical augmentation using either resorbable or titanium-reinforced barrier membranes. The authors observed minimal differences between groups, with an adjusted difference of only 0.15 mm, without statistical significance, indicating that the regenerated bone remained relatively stable over time.

In addition, histological and histomorphometric studies have provided further information on the quality of the regenerated bone. In the study by Cucchi et al. [16], histomorphometric analysis revealed that a significant proportion of the regenerated area was composed of newly formed bone, confirming that regeneration achieved through GBR does not merely reflect the presence of graft material, but rather the formation of vital bone tissue.

Overall, the studies included in this review indicate that titanium-reinforced d-PTFE membranes were associated with clinically relevant vertical bone gains, generally in the range of 4 to 5 mm, values considered clinically relevant for implant placement in atrophic alveolar ridges. Furthermore, these results demonstrate that the achieved bone gains can remain stable over time, reinforcing the clinical effectiveness of this approach in guided bone regeneration procedures.

### 4.2. Comparison Between d-PTFE-Ti Membranes and Collagen-Based Resorbable Membranes

The comparison between non-resorbable dense polytetrafluoroethylene membranes reinforced with titanium (d-PTFE-Ti) and resorbable collagen-based membranes has been widely investigated in the literature, as both are frequently used in guided bone regeneration procedures. The studies included in this review suggest that both approaches can achieve similar clinical outcomes in terms of bone regeneration, although they differ regarding graft stability and certain biological characteristics.

Several clinical trials have directly compared these two approaches in vertical bone augmentation procedures. In the study by Merli et al. [19], which evaluated changes in bone levels after vertical augmentation using either resorbable barriers or titanium-reinforced barriers, no statistically significant differences were observed between groups after a 6-year follow-up. The adjusted difference in bone level change was only 0.15 mm, indicating that both membranes were able to maintain stable outcomes over time.

Similar results were observed in studies assessing vertical bone regeneration associated with implant placement. In the randomized clinical trial conducted by Cucchi et al. [13], guided bone regeneration using d-PTFE-Ti membranes was compared with titanium meshes covered with collagen membranes. The authors reported similar vertical bone gains between both groups, demonstrating that both regenerative strategies can be effective in restoring atrophic alveolar ridges. However, the group treated with d-PTFE-Ti membranes showed a lower surgical complication rate, suggesting a potential clinical advantage of this approach.

These results were confirmed in the subsequent one-year follow-up study conducted by the same research group [14]. In this work, vertical bone gains were again comparable between d-PTFE-Ti membranes and titanium meshes associated with collagen membranes. In addition, hard and soft tissues showed good stability after one year, with peri-implant bone loss of less than 1 mm, indicating that both techniques were capable of providing adequate conditions for implant rehabilitation.

The long-term stability of the outcomes was also demonstrated in the three-year follow-up study by Cucchi et al. [15]. The authors observed that both d-PTFE-Ti membranes and titanium meshes covered with collagen membranes maintained stable vertical bone gains over time, with no significant differences between groups. These findings suggest that, from a regenerative effectiveness perspective, both approaches can be considered predictable.

In addition to clinical parameters, some studies also assessed the quality of the regenerated bone through histological and histomorphometric evaluation. In the study by Cucchi et al. [13], which compared guided bone regeneration using non-resorbable membranes with resorbable membranes associated with titanium meshes, no statistically significant differences were observed in the proportion of newly formed bone between groups. However, the PTFE membrane group showed a slightly higher proportion of newly formed bone, while the titanium mesh and collagen membrane group exhibited a slightly greater total bone area and a lower amount of soft tissue. According to the authors, this difference may be related to improved revascularization and mineralization of the graft associated with resorbable collagen membranes.

Other studies included in this review also directly compared regenerative approaches involving collagen membranes. In a recent randomized clinical trial conducted by Urban et al. [17], which evaluated the impact of collagen membrane use in vertical augmentation procedures with titanium-reinforced PTFE meshes, the results showed that the additional use of collagen membranes may contribute to better soft tissue protection and graft stabilization.

The studies included in this review indicate that both d-PTFE-Ti membranes and resorbable collagen membranes can provide comparable regenerative outcomes in terms of vertical bone gain. However, non-resorbable membranes offer certain advantages related to space maintenance and mechanical stability, whereas collagen membranes may promote better biological integration and graft revascularization. Therefore, the choice between these two approaches may depend on the characteristics of the bone defect, the surgical technique used, and the clinician’s experience.

The broader literature also supports an individualized approach to membrane selection. Titanium meshes and titanium-reinforced barriers are particularly relevant when rigid space maintenance is required, whereas minimally invasive concepts in vertical ridge augmentation aim to reduce surgical morbidity without compromising regenerative stability [23,24]. Recent reviews further support the clinical relevance of GBR for implant rehabilitation, while emphasizing that outcomes remain influenced by defect morphology, surgical design, implant-related factors, and the management of the soft tissues [25,26].

### 4.3. Complications Associated with d-PTFE-Ti and Resorbable Membranes

Although guided bone regeneration can provide clinically relevant regenerative outcomes, vertical bone augmentation procedures may be associated with different types of complications, particularly membrane exposure, infection, or partial failure of regeneration. The literature analyzed in this review shows that both non-resorbable titanium-reinforced d-PTFE membranes and resorbable collagen membranes present relatively low complication profiles, although some differences have been observed between the two biomaterial types.

Recent systematic reviews reinforce that wound dehiscence, membrane exposure, infection, and partial regenerative failure remain central complications of oral bone regeneration procedures [27,28,29]. Their clinical consequences may vary according to the timing and extent of exposure, the type of membrane, and the management strategy. Experimental developments in wound-closure materials also reflect the continuing need to improve soft-tissue stability and antibacterial protection in GBR procedures [30]. Accordingly, complication rates should be interpreted together with defect complexity, surgical technique, and membrane characteristics rather than as isolated indicators of the superiority of one biomaterial [29].

One of the most frequently reported complications in guided bone regeneration is early membrane exposure, which may occur during the healing period due to soft tissue tension or wound dehiscence. The study conducted by Cucchi et al. [13], which compared d-PTFE-Ti membranes with titanium meshes covered by collagen membranes, showed similar healing complication rates in both groups. However, a lower surgical complication rate was reported in the d-PTFE-Ti group, suggesting that this type of membrane may exhibit a more favorable clinical behavior.

Similar results were observed in the subsequent one-year follow-up study conducted by the same group [14]. In this work, the authors found that complications were comparable between both groups, with no statistically significant differences in postoperative complication rates. Furthermore, the stability of peri-implant tissues observed after one year of follow-up indicated that any initial complications did not significantly compromise the final regenerative outcome.

The long-term analysis performed by Cucchi et al. with a three-year follow-up [15] similarly confirmed that complication rates associated with both regenerative approaches remained relatively low. The authors observed that both d-PTFE-Ti membranes and titanium meshes combined with collagen membranes allowed predictable clinical outcomes, with no significant differences in complication incidence over the follow-up period.

Other studies have also evaluated the occurrence of complications associated with regenerative procedures. The randomized clinical trial conducted by Giragosyan et al. [20], which compared d-PTFE-Ti membranes with customized 3D-printed titanium meshes, showed similar complication rates, indicating that PTFE-based membranes remain a valid option even when compared with more recent technologies.

Non-resorbable membranes offer the advantage of better space maintenance due to their higher rigidity and structural stability. However, these membranes generally require a second surgical procedure for removal, which may increase treatment morbidity. In contrast, resorbable collagen membranes provide better biological integration and do not require surgical removal, although they may exhibit a reduced ability to maintain space in larger bone defects.

The choice between d-PTFE-Ti membranes and resorbable membranes should consider not only the regenerative potential of the material, but also clinical factors such as defect morphology, the need for space maintenance, and the surgeon’s experience. Overall, the studies included in this review indicate that both types of membranes can be used with acceptable levels of safety and clinical predictability in guided bone regeneration procedures.

Although peri-implantitis defects represent a different clinical indication from vertical ridge augmentation, network meta-analytic evidence also illustrates the broader heterogeneity of GBR protocols and reinforces the importance of matching regenerative strategies to defect configuration and treatment objectives [31].

### 4.4. Limitations

Despite our results, several methodological limitations must be considered when interpreting the data presented in this review. One of the main limitations relates to the heterogeneity of the outcomes assessed across the included studies. While some authors primarily evaluated vertical bone gain after guided bone regeneration [13,14,15,19], others focused on peri-implant marginal bone level changes over time [18,19]. This diversity of endpoints makes direct comparison between studies difficult and limits the ability to draw definitive conclusions regarding the superiority of one membrane type over another.

Another important limitation relates to differences in bone measurement methods. In the included studies, assessment of bone regeneration was performed using various approaches, including intraoperative clinical measurements, radiographic or tomographic analyses, and in some cases histological or histomorphometric evaluations [16]. These methodological variations may introduce differences in measurement accuracy and further complicate direct comparison of results across studies.

In addition, follow-up periods varied considerably among the included studies, which represents a relevant limitation in comparing outcomes. While some studies reported only short-term results with follow-up periods of approximately 6 months to 1 year [13,17,18], others evaluated medium-term outcomes with follow-up periods of around 1 to 3 years [14,15]. Furthermore, some studies provided long-term follow-up data of up to 6 years [19]. This temporal variability may influence the interpretation of the results, as additional bone changes particularly remodeling and marginal bone loss may occur after the initial healing period, making direct comparison between studies more challenging.

One other significant limitation relates to the relatively small sample sizes in several of the included studies. Many randomized clinical trials in the field of guided bone regeneration involve a limited number of patients, often fewer than fifty participants [13,14,15]. Although these studies provide valuable information on the effectiveness of regenerative techniques, small sample sizes may reduce the statistical power of the analyses and limit the generalizability of the findings to broader populations.

Finally, it should also be considered that some of the included studies evaluated combinations of different biomaterials and surgical techniques, such as the use of titanium meshes associated with collagen membranes or the use of bone grafts from different sources [13,14,15,17]. These variations may influence the observed regenerative outcomes and make it difficult to isolate the specific impact of the membrane type used.

Although the studies analyzed provide relevant evidence on the effectiveness of d-PTFE-Ti membranes and resorbable collagen membranes in guided bone regeneration procedures, these methodological limitations should be considered when interpreting the results. Future research with larger sample sizes, more standardized methodological protocols, and longer follow-up periods should be considered in order to clarify more definitively the relative role of these biomaterials in vertical bone augmentation.

## 5. Conclusions

In line with the primary objective of this review, the available evidence suggests that titanium-reinforced d-PTFE membranes can achieve clinically relevant vertical bone gains. Overall, the vertical bone augmentation values reported in the included studies ranged from approximately 4 to 5 mm, enabling implant rehabilitation in previously atrophic alveolar ridges. In addition, the available follow-up data suggest that the regenerated bone can remain relatively stable over time, with low levels of marginal bone loss reported in several studies.

In accordance with the secondary objective, the available evidence suggests that both d-PTFE-Ti membranes and resorbable collagen-based approaches can provide similar regenerative outcomes. Non-resorbable membranes may offer greater mechanical stability and space maintenance, whereas collagen membranes may provide advantages related to biological integration. Regarding complications, the available evidence does not support a definitive overall advantage of either approach.

The findings suggest that guided bone regeneration using titanium-reinforced d-PTFE membranes can provide clinically relevant outcomes for vertical alveolar ridge augmentation. However, the heterogeneity of the included studies particularly in terms of evaluated outcomes, measurement methods, follow-up periods, and sample sizes limits direct comparisons and the generalization of results. Therefore, future research with more standardized methodologies, larger sample sizes, and longer follow-up periods is required to confirm these findings and further optimize the clinical application of these techniques.

## Figures and Tables

**Table 1:** PICOS’ strategy.

Population	Patients with vertical atrophy of the alveolar ridge.
Intervention	Guided bone regeneration with d-PTFE-Ti membranes.
Comparison	Collagen-based resorbable membranes.
Outcomes	Vertical bone gain, bone stability, and complications.
Study Design	Randomized Controlled Trials and cohort studies

**Table 2:** Inclusion and exclusion criteria.

Inclusion Criteria	Exclusion Criteria
Studies published from 2014 onwards	Studies published before 2014
Studies published in English, Portuguese, French, and Spanish.	Animal studies
Clinical studies conducted in humans.	Systematic reviews.
Studies including patients with bone defects or alveolar ridge atrophy requiring bone regeneration procedures for subsequent implant rehabilitation.	Studies that did not use PTFE membranes or resorbable collagen membranes.
Studies evaluating guided bone regeneration (GBR) procedures.	Studies that did not present clinically relevant data related to bone regeneration.
Studies using non-resorbable polytetrafluoroethylene (PTFE) membranes, particularly titanium-reinforced d-PTFE, or resorbable collagen-based membranes.	Studies focused exclusively on horizontal bone augmentation without relation to vertical regeneration.
Studies conducted in the context of implantology, provided they included relevant data on bone regeneration.	

**Table 3:** Risk of bias for Randomized Controlled Trials.

	Question	1	2	3	4	5	6	7	8	9	10
Study/Risk											
[13]: low		Y	Y	Y	N	N	Y	Y	Y	Y	Y
[14]: low		Y	Y	Y	N	N	Y	Y	Y	Y	Y
[15]: moderate		Y	Y	Y	N	N	Y	Y	Y	N	Y
[16]: low		Y	Y	Y	Y	Y	Y	Y	Y	N	Y
[17]: moderate		Y	Y	Y	N	N	N	UN	Y	Y	Y
[18]: low		Y	Y	Y	UN	N	Y	Y	Y	Y	Y
[19]: low		Y	Y	Y	N	N	Y	Y	Y	Y	Y
[20]: low		Y	Y	Y	Y	N	Y	Y	Y	Y	Y

1. Was true randomization used for assignment of participants to treatment groups? 2. Was allocation to treatment groups concealed? 3. Were treatment groups similar at the baseline? 4. Were participants blind to treatment assignment? 5. Were those delivering the treatment blind to treatment assignment? 6. Were treatment groups treated identically other than the intervention of interest? 7. Were outcome assessors blind to treatment assignment? 8. Were outcomes measured in the same way for treatment groups? Were outcomes measured in a reliable way? 9. Was follow-up complete and if not, were differences between groups in terms of their follow-up adequately described and analysed? 10. Were participants analysed in the groups to which they were randomized? Was appropriate statistical analysis used?

**Table 4:** Risk of bias in cohort studies.

	Question	1	2	3	4	5	6	7	8	9
Study/Risk										
[21]: low		Y	Y	Y	Y	Y	Y	UN	N	Y

1. Were the two groups similar and recruited from the same population? 2. Were the exposures measured similarly to assign people to both exposed and unexposed groups? 3. Was the exposure measured in a valid and reliable way? Were confounding factors identified? 4. Were strategies to deal with confounding factors stated? 5. Were the groups/participants free of the outcome at the start of the study (or at the moment of exposure)? 6. Were the outcomes measured in a valid and reliable way? Was the follow-up time reported and sufficient to be long enough for outcomes to occur? 7. Was follow-up complete, and if not, were the reasons for loss to follow-up described and explored? 8. Were strategies to address incomplete follow-up utilized? 9. Was appropriate statistical analysis used?

## Data Availability

The data can be accessed by contacting the corresponding author.

## Supplementary Materials

The following supporting information can be downloaded at: https://www.mdpi.com/article/10.3390/jfb17080353/s1, Table S1: PRISMA checklist.

## Abbreviations

GBR	Guided Bone Regeneration
VBG	Vertical Bone Gain
MBL	Marginal Bone Loss
d-PTFE	Dense Polytetrafluoroethylene
d-PTFE-Ti	Dense Polytetrafluoroethylene reinforced with Titanium
e-PTFE	Expanded Polytetrafluoroethylene
Ti	Titanium
RCT	Randomized Controlled Trial
VRA	Vertical Ridge Augmentation
CBCT	Cone Beam Computed Tomography
